# The Barriers to Universal Health Coverage in India and the Strategies to Address Them: A Key Informant Study

**DOI:** 10.5334/aogh.4120

**Published:** 2023-10-09

**Authors:** Anuska Kalita, Noah Carton-Rossen, Linju Joseph, Deepshikha Chhetri, Vikram Patel

**Affiliations:** 1 Harvard T.H. Chan School of Public Health, USA; 2 Achutha Menon Centre for Health Science Studies, India; 3 University of Birmingham, UK; 4 Samagra, India; 5 London School of Hygiene and Tropical Medicine, UK; 6 Harvard Medical School, USA

**Keywords:** universal health coverage, health system, health policy, India

## Abstract

**Background:**

India has adopted several policies toward improving access to healthcare and has been an enthusiastic signatory to several global health policies to achieve Universal Health Coverage (UHC). However, despite these policy commitments, there has been limited success in realizing these goals. The COVID-19 pandemic has highlighted the urgent need for health system re-design and amplified the calls for such reforms.

**Objectives:**

We seek to understand the views of a diverse group of policy actors in India to address the following research questions: what are the (i) conceptualizations of UHC, (ii) main barriers to realizing UHC, and (iii) policy strategies to address these barriers.

**Data and Methods:**

We collected data through in-depth interviews with 38 policy actors from diverse backgrounds and analyzed using the Framework Method to develop themes both inductively and deductively using the Control Knob Framework of health systems.

**Findings:**

There was congruence in the conceptualization of UHC by policy actors. Quality of care, equity, financial risk protection, and a comprehensive set of services were the most commonly cited features. The lack of a comprehensive systems approach to health policies, inadequate and inefficient health financing mechanisms, and fragmentation between public and private sectors were identified as the main barriers to UHC. Contrasting views about specific strategies, health financing, provider payments, organization of the delivery system, and regulation emerged as the key policy interventions to address these barriers.

**Discussion and Conclusion:**

This is the first systematic examination of a diverse set of policy actors’ problem analyses and suggestions to advance UHC goals in India. The study underscores the need to recognize the complex and interlinked nature of health system reforms and initiate a departure from path-dependent vertical interventions to bring about transformative change.

## Background

India has adopted several policies, both at the national and state levels, toward improving access to healthcare. It has also been an enthusiastic signatory to several global health policies, particularly to achieving Universal Health Coverage (UHC). However, despite repeated assertions by successive governments, there has been limited success in realizing these goals. India has made some remarkable progress on population health indicators – infant mortality rate has decreased from 79 to 35, and maternal mortality from 437 to 97 between 1992 and 2020 [1,2]. The country has significantly improved physical access to services – nearly 96% people utilize healthcare when ill, 84% of children are fully vaccinated in 2020 compared to 35% in 1992, and institutional births have seen a 63-percentage point increase during the same time period [1,2]. India’s UHC service index score has improved in the last decade [3], but it still ranks poorly on effective UHC coverage with a score of 47, compared to its neighbors like Bangladesh (54), and emerging economy peers like China (70), Brazil (65), and Mexico (61) [4]. Specifically, financial risk protection continues to be poor, with 57% of total health expenditures spent by households out-of-pocket, socioeconomic and geographical inequities persist, and limited evidence shows that quality of care is disconcertingly poor [1,5,6]. The alarming toll of the COVID-19 pandemic has further highlighted the urgent need for health system re-design and has amplified the calls for such reforms from different stakeholders [7].

The Lancet Citizens’ Commission on Reimagining India’s Health System (the Commission), launched in 2021, is an ambitious cross-sectoral endeavor to identify the transformative reforms needed to achieve UHC over the next decade. Taking a comprehensive and participatory approach, the Commission seeks to bring together diverse stakeholder perspectives to address the complex challenges facing India’s health system [8]. The study described in this paper is one such effort of the Commission in which we analyze the perspectives of leading policy actors on the barriers that have impeded progress toward UHC and the policy strategies which may address them. Specifically, we seek to understand the views on the following research questions: what are the (i) conceptualizations of UHC, (ii) main barriers to realizing UHC, and (iii) policy strategies to address these barriers?

Borrowing from the WHO’s Alliance for Health Systems and Policy Research [9], we operationally define policy actors as those who: (i) have specific responsibility for developing formal policies in the public or private sectors, including those outside the health sector working on health-influencing policies, and international agencies and organizations, (ii) influence how policies are translated into practice (such as middle managers, health workers, patients, and citizens), and (iii) seek to influence the formal policy process (such as civil society groups or interest groups). Our findings are intended to inform the recommendations of the Lancet Citizens Commission and, to the best of our knowledge, it is the first study to systematically analyze policy actors’ perceptions about barriers and strategies to achieve UHC in India.

## Data and Methods

### Sample

Our sample includes 38 policy actors, classified into two types: (i) individuals from research/academia, civil society, and the private sector who have provided technical advisory support on UHC-related reforms and (ii) civil servants who have been involved in designing or implementing health policies at the national and state levels. We used convenience and snowball sampling. First, we compiled a list of the Commissioners of the Lancet Commission, authors of key policy documents and members of government task forces, and senior civil servants at the national and state health departments. Then, during our interviews, we utilized snowball sampling by asking our respondents for recommendations of other policy actors to interview. Thirty-eight out of 43 policy actors initially approached for interviews consented to participate in the study. Two declined to participate due to concerns about potential conflicts between their views and their formal institutional positions; three could not participate due to scheduling conflicts. We stopped further sampling after reaching thematic saturation.

### Data collection

These 38 in-depth interviews using a semi-structured guide were conducted virtually via Zoom. The interview guide comprised open-ended questions followed by probes. The interview guide included topics on stakeholders’ definitions of UHC, their perceptions of the main barriers to achieving UHC, and their perceptions of future reforms India should adopt to advance UHC. Each interview lasted approximately 60 minutes. Thirty-five respondents gave permission to video and audio record the Zoom interviews. Audio files were transcribed verbatim. Three interviews were not recorded based on the interviewee’s request, but detailed notes were taken.

### Data analysis

Data were analyzed using the Framework Method, which allowed a combined approach to analysis, enabling themes to be developed both inductively from the accounts (experiences and views) of research participants and deductively from existing literature [10]. The Framework Method originated in large-scale social policy research but is becoming an increasingly popular approach in multi-disciplinary health research [11]. For the deductive part of the analysis, we used the Control Knob Framework (CKF) developed by Roberts and colleagues [12]. The CKF is based on a set of relationships in which certain structural components (the means) and their interactions are connected to the goals the health system intends to achieve (the ends). The means, comprised of five policy areas or “control knobs” (*health financing; strategic purchasing and provider payments; organization of the delivery system; regulation; and persuasion*), lead to the ends (three intermediate goals: *access, quality*, and *efficiency*), and three final goals (*health status, financial risk protection*, and *citizen satisfaction*). For all goals, the framework considers both level of performance (compared to various benchmarks) and equity dimensions. Compared to other health systems frameworks, the CKF is distinguished by its action orientation and policy outcomes-based logic. In short, the CKF focuses on actions that can be taken by policy actors to improve performance in measurable ways through causal linkages between health system components and outcomes. These features of the CKF make it particularly relevant for our study, where we seek to explore how our policy actors conceptualize health system goals – in this case, UHC – and what “control knobs” or policy levers they recommend to achieve these goals.

Based on the Framework Method, our data analysis process involved the following steps:

- The audio recordings were transcribed into text files, and the transcripts were compared with the audio files to ensure their veracity.
- The transcripts (or notes for the three interviews which were not recorded) were analyzed to develop initial codes in two ways: with a deductive approach using the Control Knob Framework and an inductive approach. This initial coding was undertaken independently by four researchers to increase inter-coder reliability.
- Iterations were made to the codes to align coding by two researchers and develop categories of codes.
- Themes were developed by interrogating code categories through comparison between and within cases, and different code categories were grouped under each theme or sub-theme.
- The final thematic analysis focused on interpreting similarities and differences amongst policy actors on the major themes of the research.

## Findings

**Figure 1:**
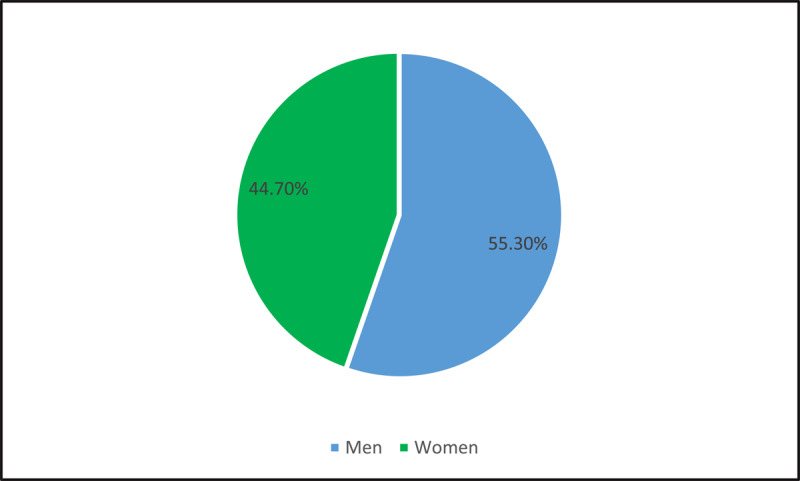
Characteristics of the sample: Respondents by their gender.

**Figure 2:**
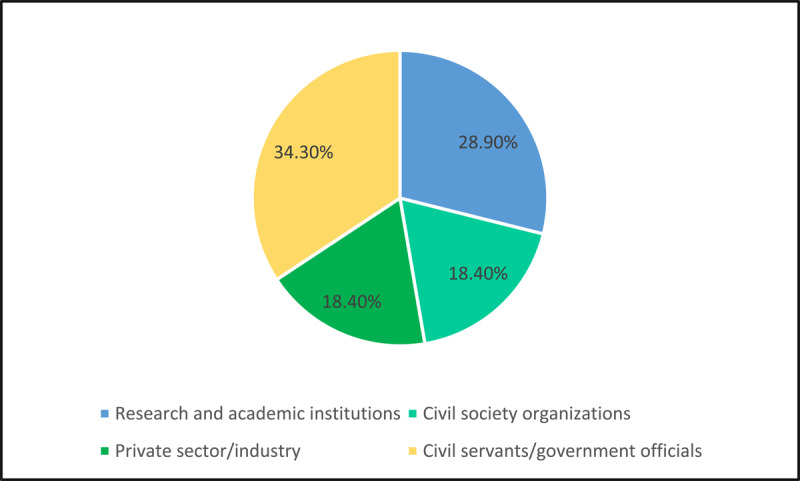
Characteristics of the sample: Respondents by their primary professional affiliations.

We interviewed 38 policy actors (17 women and 21 men). Our respondents represented four diverse sectors in health – civil servants (n = 13), members of the academy (n = 11), and equal numbers from civil society and industry (n = 7 each). Figures 1 and 2 present our sample characteristics.

We present our findings along three main themes emerging from our data: (1) the understanding and conceptualization of UHC by the policy actors, (2) the barriers to achieving UHC, and (3) the policy reforms and strategies to address these barriers.

### 1. The conceptualization of UHC

We observed a high degree of congruence in the conceptualization of UHC, closely aligned with the WHO’s definition [13]; the most commonly cited aspects being quality of care, equity, financial risk protection, and comprehensive care. Most policy actors framed UHC from a problem analysis lens, and their definitions of UHC overlapped with their perception of the key gaps in India’s progress on UHC goals. For example, quality of care was conceptualized as a core aspect of UHC, and poor quality of care was identified as one of the most significant problems facing India’s health system. Respondents concurred that India had made commendable progress on several health indicators, especially in improving physical access and utilization of services. We did not find contrasting definitions of UHC among the respondents, although some characteristics, like quality of care and equity, were emphasized more commonly than others. Table 2 presents relevant quotes for each of our sub-themes.

#### 1.1 Quality of care

The most recurrent theme was “access to quality care” as an integral aspect of UHC, and many respondents referred to the “unacceptably” poor quality of care in the Indian health system. Some respondents highlighted the nuances of poor quality in the public and private sectors – with the main concerns being under-provision, low patient trust and satisfaction in the public sector and over-provision and unnecessary care in the private sector. However, notwithstanding the overall poor quality, our data reveals that respondents commonly believed the private sector was providing higher quality care than the public sector (see Table 1, quote 1.1).

**Table 1:** Interview quotes about policy actors’ conceptualization of UHC.


SUB-THEMES	INTERVIEW QUOTES (RESPONDENT CODE IN PARENTHESES)

**1.1 Quality of care**	The goal of universal healthcare would be access to good quality healthcare services. Quality being a very important, what I would call a multiplicative factor not an additive factor. Without quality, you can provide good clinics, but you have to multiply by zero if the quality is not there and therefore you have to assume access is not there. It is like the subcenters that you see of government establishments, there is no quality at all, so they might as well not be there. (ID7, research/academic institution)

**1.2 Equity**	Equity is the most important thing in universal coverage. As I said, “last mile first.” That is equity… SDGs [Sustainable Development Goals], they call it nobody should be left behind. You know, that is the slogan for this. Ultimately…you have to reach the underprivileged, [they] are the ones who need it the most, and you have to provide services to them. That is the real litmus test for the UHC. (ID27, civil servant/government official)

**1.3 Financial risk protection**	We can’t have this kind of free for all going on where people are [paying] such high out of pocket [costs] and are being exploited…many of the private players and even public people take money from poor people. We’ve seen how they got in debt…They have spent like two-three lakhs trying to save their family members. You know mortgaging their land and that’s just not acceptable. That’s just a travesty in this day and age. We know what we have to do, we need to do it…the costs should be within [a certain] range, not explode exponentially. (ID4, civil society organization)

**1.4 Comprehensive set of services**	Universal health coverage firstly is coverage to every Indian, and every resident of the country and it should be comprehensive. Universal suggests both universalities of coverage, that means everybody is included, as well as universal in the sense that it covers all the conditions that are caused by diseases and other medical conditions. (ID26, private sector/industry)


**Table 2:** Interview quotes about policy actors’ perception of barriers to UHC.


SUB-THEMES	INTERVIEW QUOTES (RESPONDENT CODE IN PARENTHESES)

**2.1 The lack of a “systems” approach to health reform**	We are still trapped in our verticals. Like verticals of TB, verticals of maternal child health… So these all have to be integrated. That kind of discussion has to be routinely happening at the political level, political leadership level in health, as well as administrators, so that is not happening… So alignment has to be there. The second thing is that the integration, so you know, all these verticals coming together to deliver universal health care, so that has to happen. (ID24, civil society organization)

**2.2 (a) Barriers related to health financing**	It has to also do with financial accountability. How are we getting the most optimal return on our investments?… With whatever money is invested in the health sector, are we getting the best return on that investment? And the answer is no. Because a large amount of public funds are underutilized. I mean they’re sitting there in the state kitties, and they’re not used. So that’s one problem. The money that is used is not necessarily used well. So, therefore, that is another reason that you’re not getting the most optimal results and all of this is because the capacities are low, and accountability is largely absent. (ID29, research/academic institution)

**2.2 (b) need for expanded insurance coverage**	First, would be the poorest of the poor or the people who just can’t afford any spending on health care. So that would probably be the bottom 40 to 50% population that has been tried to be covered through…the PMJAY scheme [*India’s government insurance program*] of the government of India. And there’s a top 10 to 15% population of the country that…can spend out of pocket and can very well take care of their healthcare needs. Now comes the missing middle 20 to 30% plus population that remains to be covered… They would require some sort of coverage because as we see these other people from the unorganized sector, they might be workers, they might be earning a wage, they might not come into the category of poor people, but they are not so rich that they can afford the health care expenses on their own and not having any dent on their pockets. (ID33, civil servant/government official)

**2.3 (a) Barriers related to the organization of healthcare delivery**	There are plenty of instances where basic economic reasoning would need you to think that somethings should be done more with the private sector than by the public sector… So in those cases we should consider privatization. But I think those who vociferously […] oppose privatization also have a point, because the private sector in India has plenty of instances of misbehavior, taking advantage of the consumer, overcharging, over-treating, unsavory collection practices, you know contaminated medication…there’s a long list of things where the private sector is implicated… I don’t find this debate very productive because there’s…[a] similarly long list of things in the public sector where you could find problems. So there’s no point demonizing [a] way of economic organization. (ID5, research/academic institution)

**2.3 (b) Hospital-centric design of the health system and bypassing of primary care**	Our system is more robust than any other, but we need to take it to the…lower level… So preventive and promotive care, basic NCD care, and along with antenatal care some kind of population-based coverage and screening gives the promotive aspect as well as an understanding of the community… Then linking this nicely and integrating this with the secondary and tertiary care referral mechanisms which we have not succeeded in our country at all. We don’t have gatekeeping everybody goes for [a] cough and cold to the specialist in the government hospital. (ID19, civil servant/government official)

**2.3 (c) Need for task shifting**	There’s this [human resource] imbalance…how resistant India has been for delegating functions to midwives, for example, when the whole world has acknowledged, the role of midwives…the need for delegating more responsibilities and powers of treatment, diagnosis, and prescription practices from the doctors to other non-medical professions. But only India tightly holds on to the doctor led system… It is not possible to have…30,000 medical colleges and 3,000,000 doctors…it’s not possible. So you have to use a whole lot of a team approach and have more skills and marry the skills available at each level of care with the kind of disease burden that is there, rather than have a prescriptive formula. (ID21, civil servant/government official)

**2.4 Regulation, stewardship, and decentralization**	I think at the center there is definitely poor political commitment…for some reason, it’s not a winning political issue… People underestimate their likelihood of falling sick and needing health care…or at least serious healthcare, you know, hospital stays, or expensive surgeries… And so the masses apparently don’t reward this as a political issue and that translates into a lack of political will at the center. It’s also expensive and I think…we have to have a serious discussion about the trade-off. If you want to provide health care coverage to anything close to universal, you know, it’s going to be expensive, and obviously, in a country like India there, that means that money that can be used for other things will have to be used for healthcare. (ID22, research/academic institution)


#### 1.2 Equity

Equity was highlighted as another key characteristic of UHC. Policy actors most commonly mentioned the need to achieve equity between high and low-income groups. The need to bridge geographical inequities between and within states and tailor UHC efforts to address state-specific and rural-urban inequities emerged as an important sub-theme, while others also emphasized the inequities among different caste and indigenous groups (see Table 1, quote 1.2).

#### 1.3 Financial risk protection

The other commonly recurring theme was poor financial risk protection (FRP), i.e. people facing financial hardships in accessing high-quality healthcare. Respondents expressed the need for UHC to ensure FRP among India’s population. Some respondents emphasized the over-charging and predatory practices by private providers causing financial distress among patients, often citing examples from the COVID-19 pandemic. Other interviewees discussed the nuances of out-of-pocket expenses (OOPE) and catastrophic health expenditures (CHE), even within the public sector which theoretically provides “free” services (see Table 1, quote 1.3).

#### 1.4 Comprehensive set of services

UHC was perceived as a “comprehensive set of services,” and most respondents conceptualized UHC as covering the different levels of care and different needs and health conditions, including prevention, primary, secondary, tertiary, and rehabilitative care. The importance of primary care and inclusion of often-neglected conditions like non-communicable diseases and mental health were often mentioned as critical aspects of “true” UHC (see Table 1, quote 1.4).

### 2. Barriers to achieving UHC

This theme includes the health system-related barriers that policy actors identified as impediments to India’s progress on UHC goals. The lack of a comprehensive approach to health reforms emerged as an overarching theme underlying the barriers in health financing, fragmentation of healthcare delivery, poor regulation, and failures of decentralization. Table 2 presents selected interview quotes for each of the sub-themes.

#### 2.1 The lack of a systemic approach to health reforms

The lack of a comprehensive health systems approach in health policies emerged as an overarching barrier to achieving UHC in India. Policy actors perceived that health policies had been vertical, often disease-specific programs, and fragmented instead of taking a comprehensive “systemic” approach. The focus on increasing physical access and building infrastructure over other health systems goals like improving quality and efficiency, the failure to envision the interlinkages among different parts of the health system, and the failure to account for the roles of the public and private sectors, both in financing and delivery of healthcare, were perceived to be major challenges to India’s progress on UHC. This theme of a vertical or fragmented versus a systemic approach underlies the other barriers in health financing, organization, and regulation presented below (see Table 2, quote 2.1).

#### 2.2 Barriers related to health financing

Health financing emerged as the second major barrier to UHC. Policy actors perceived challenges and shortcomings in all three main functions of health financing – the amount of funds allocated to health, how funds are pooled, and how services are purchased. Most interviewees mentioned inadequate government health expenditures as a major constraint to achieving UHC. Respondents acknowledged that low government health expenditure (GHE) was primarily due to limited fiscal space to increase health budgets and competing priorities like food and nutrition security, infrastructure, and education. However, there were contrasting opinions. One of the key sub-themes in our data was the inefficient utilization of existing funds. Some policy actors perceived that total health expenditures, or even GHE, in some Indian states were adequate, but low utilization capacities in the public sector and allocative inefficiencies were the more pressing barriers to UHC. In some instances, these inefficiencies act as deterrents (or justifications) for governments against increasing funding (see Table 2, quote 2.2 (a)).

Inadequate prepayment, pooling, and poorly designed provider payment mechanisms (PPMs) were seen as interlinked sets of barriers to UHC. While government health insurance programs currently cover 40% of the population, the middle-income households that constitute the majority of India’s population were excluded from government schemes targeted mostly at low-income groups and commercial health insurance plans catering to high-income consumers. Additionally, policy actors highlighted that most insurance plans covered only hospitalizations and excluded outpatient care and medication, although the latter contributed to most of OOPE. Respondents expressed that the current fee-for-service (FFS) payments at the point of care increased the risk of catastrophic and impoverishing health expenses (CHE and IHE), often leading to foregone or delayed care for a significant proportion of India’s population. The current PPMs, like line-item budgets and salaries in the public sector, were seen as causing under-provision of care and creating an inefficient public sector, while FFS used in the private sector were perceived as causing price gouging and irrational and unnecessary interventions. Policy actors mentioned how both sets of PPMs created distorted incentives for the entire health system, leading to poor quality or low-value care and high and wasteful healthcare expenses. Respondents perceived limited coverage of government and commercial insurance programs, both in terms of population coverage and benefits packages, as exacerbating the problem of poor financial risk protection (FRP) (see Table 2, quote 2.2 (b)).

#### 2.3 Barriers related to the organization of healthcare delivery

Policy actors identified barriers within three sub-themes – fragmentation across the health system (especially between the public and private sectors and among different levels of care), selective primary care and hospital-centric care, and challenges with human resources for health (HRH). Respondents expressed that the fragmentation or “fracture” between the public and private sectors in healthcare has led to inequities in access to high-quality healthcare, with those who can afford private healthcare having access to better quality care than those who depend on public facilities. Additionally, the lack of coordination between the two sectors has resulted in misalignment of provider incentives, inefficient allocation of resources, and duplication of efforts, leading to low-value care. The historically limited focus of Indian health policies on the public sector or only large corporate hospitals, while ignoring the large heterogeneous mix of providers like private pharmacies, solo practitioners, and smaller private health facilities, was identified as a major reason behind this fragmentation (see Table 2, quote 2.3 (a)).

Respondents perceived the Indian health system as hospital- and physician-centric, focusing on curative, doctor-led, care instead of comprehensive primary care. The public sector’s focus on building and delivering care through large hospitals and providing selective primary care for infectious diseases and maternal and child health were seen as key barriers to realizing UHC. Respondents also expressed that this skewed design led to the underutilization of most public sector primary care facilities, where people “bypassed” these and sought care at hospitals even for minor illnesses. This, in turn, exacerbated inefficiencies across the system and led to delayed care, foregone care, and unmet needs (see Table 2, quote 2.3 (b)).

Related to this, policy actors highlighted that skewed HRH planning and allocations, focusing on merely increasing the number of physicians instead of concentrating on role definitions, were a barrier to UHC. Respondents raised that resistance by physician associations to task shifting that would allow non-physician personnel and practitioners of Indian systems of medicine to provide a large proportion of healthcare impeded the goal of improving access to universal primary care. Some respondents listed inequitable distribution of HRH, poor quality of training across personnel, weak support structures, and low motivation as challenges. Attrition of qualified providers from the public to private sectors, and from India to other countries, due to better incentives, was another challenge in HRH (see Table 2, quote 2.3 (c)).

Some respondents touched upon the vicious cycle of under-investment and underutilization in the public sector leading to further degradation in its quality of services, expressing that the private sector attracted more financial and qualified human resources, often “hollowing out” the public sector. Equipped with better resources, the former attracted the majority of the middle class as patients, while the latter largely catered to the poor – a group that had far lower socioeconomic power and “voice” to demand better health services. On the other hand, other respondents perceived misaligned provider incentives, lack of accountability mechanisms, and flawed financing as deepening the fragmentation and inequities.

#### 2.4 Regulation, stewardship, and decentralization

Another major set of barriers to UHC was related to regulation, stewardship, and decentralization. The need for good governance practices to increase accountability and efficiency in the public sector and the need to regulate the private sector to address overcharging and unnecessary care were near-universal themes in our data. The need for the government to play a stewardship role instead of being another “provider” or a minor “financier” of healthcare in the country was highlighted by some respondents. Although less commonly mentioned, weak state capacity to enforce regulations and steward a large, heterogeneous, and powerful private sector, and corruption in the accreditation of healthcare providers and medical colleges, were acknowledged as challenges by some interviewees. Related to this, policy actors mentioned that although health is legislatively the states’ mandate in India, weak capacity at decentralized levels to design and implement UHC reforms led to such reforms being centralized and implemented in national missions, which were often not responsive to local contextual realities. Finally, a barrier raised by a few respondents, was the insufficient political priority and commitment to health by successive governments as well as by the public as an electoral issue (see Table 2, quote 2.4).

### 3. Strategies for addressing these barriers to UHC

To address barriers in UHC, policy actors offered strategies in four key areas – health financing, healthcare delivery organization, use of digital technologies, and regulation and decentralization. Table 3 presents relevant interview quotes about each of these sub-themes.

**Table 3:** Interview quotes about policy actors’ suggestions for strategies for addressing barriers to UHC.


SUB-THEMES	INTERVIEW QUOTES (RESPONDENT CODES IN PARENTHESES)

**3.1 (a) Strategies for health financing -inefficiencies in resource use**	First and foremost is finance. It’s the lack of actual investment… government has borrowed money you know, but for it to actually translate into investments into primary secondary care, and to certain extent tertiary care. It’s a long road ahead…are they being appropriately allocated?…are they being efficient? There’s a lot of wastage and leakage, of course, there’s a lot of wastage, but first we have to invest… This is like two opposite ends of the spectrum. One says, “no, no, you stop the wastage” and [one says,] “no, no, you have to invest more.” So there is that problem. And I am not arguing that there is no wastage or leakage. But we’ve never tried…to say, “let them be self-sufficient.” (ID28, civil servant/government official)

**3.1 (b) Strategies for health financing -pooling and catastrophic payments**	Every country has very very desperately poor people. They should be offered health care for a small premium by the government. And for people who can afford to pay, there should be different tiers of…health insurance with different premiums. So essentially, hospitals shouldn’t have a cash counter. There has to be a financial intermediary paying for the healthcare and there should be a culture of paying a small amount of money by everyone, every family when they are all well so that when somebody is unwell they don’t need to pay… So essentially all I’m trying to say, in a nutshell, is that every country should concentrate on creating a financial intermediary…but if you say that, “government has to offer health care, it has to be free,” that is a very utopian dream. (ID25, private sector/industry)

**3.1 (c) Strategies for health financing – insurance premium collection**	They should create a mechanism to collect the premium from the members. Now, when you collect the premium from the members, there is again a mental block… If you…tell them that you have to pay 50 rupees or 100 rupees…every month to this entity. Most people won’t bother. But if you tell them… Okay, you need the electricity connection to your house. Okay, with your electricity bill we are going to charge you extra 100 rupees or 200 rupees, that they don’t mind… Okay, so that is one option. Another one is a mobile phone. Every month they have to pay the mobile bills… Otherwise, the cost of collecting money will be more than the cost of the premium. (ID25, private sector/industry)

**3.1 (d) Strategies for health financing – expand insurance**	PMJAY [India’s GHI program] provides insurance for…40% of India’s population and not the entire population. Secondly, it is only for secondary and tertiary care. So what is universal health?… PMJAY doesn’t do either. It’s not all citizens, and it is not all [care]… And because it doesn’t cover primary care that whole lack of quality out-of-pocket expenditure is spent… So that’s a large part of the middle-income groups of India are actually not covered by any insurance, because PMJAY will cover your 40% and then the higher levels buy their own private insurance, but there is this huge portion in the middle who actually are not covered at all. (ID29, research/academic institution)

**3.1 (e) Strategies for health financing – essential services package**	Universal health coverage in India’s context should be…that everyone, rich or poor, in whichever part of the country they live in,…be eligible for certain essential services…you know those which are absolutely life-saving. I don’t think India is in a fiscally in a position to be able to ensure and guarantee that all services under health, complete total health care,… can be provided free of cost…it’s just not possible. And so I think even if India could in the short run, limit itself to say essential health services, and essential can be well defined in accordance with the disease burden and the most common ailments and those which are contributing to disproportionately higher out-of-pocket expenditures…there has to be…fiscal affordability in mind. (ID21, civil servant/government official)

**3.1 (f) Strategies for health financing – provider payment mechanisms**	At the level of policy and programming, I think we need a conceptual shift from disease-based programming to per capita based or population-based programming. Universal health coverage is about a unit of population, whether it’s a city, a ward, a village district, whatever it might be. And the goal is that you are improving the health of the entire population in an equitable way, rather than basically treating disease one, disease two, disease three. (ID1, research/academic institution)

**3.2 (a) Strategies for organization of the healthcare delivery system**	It’s a healthy competition. Fifty percent of the market has to be with us in the public sector, 50% “you do what you want”, leave it to the private sector…with the assumption that you cater to the well-off and the rich who can afford you. But at least 50% we provide in the public sector. And these both…also see a strong public sector as essential to have a healthy private sector for keeping the prices under control. So [if] you remove the public sector totally or weaken it,…then you’ll find the private sector becoming extremely predatory and exploitative resorting to irrational care, resorting to unethical conduct, and all the ills of our market, commodifying their product… Only Kerala and Tamil Nadu are trying to find their balance… In any other state, it is either brazenly pro-private…or it is…trying to be strengthening the public sector and not necessarily working with the private sector to come along… It’s not going to be possible for the government to provide all services. It is not possible for us to do away with the private sector, but it is certainly possible for us to come up with appropriate policies to see that the private sector is a responsible partner and not exploitative. (ID21, civil servant/government official)

**3.2 (b) Strategies for organization of the healthcare delivery system**	I suggested to them “get out of primary care.” You cannot execute it if you don’t have the money. Even the ASHA worker…was able to deliver it to only three and a half per cent of pregnant women. So she might as well not be there… Right so I said to them to focus on the two other things. One is public health. Get your vaccinations done, think about you know social determinants, think about one health, think about that, think about zoonoses, surveillance…education, etc. And then we’ll do secondary. (ID7, research/academic institution)

**3.2 (c) Strategies for organization of the healthcare delivery system – human resources for health**	It’s these grassroots workers, community-based workers who can make that difference. But they need training, they need supervision, they need motivation and holding, and the systems for them have to be strengthened, because it was formed in 1970… They’ve been umpteen reports, which have said [this], none of those recommendations has been implemented. (ID8, civil society organization)

**3.3 (a) Using digital technologies and data systems**	There are many applications of digital health. Some of them are very, very useful. For instance, teleconsultation between a medical officer and a specialist is very, very good…so that is fantastic use of digital health… [But] this is not happening. First of all, in the village, you won’t have connectivity… So I think to choose technologies and the use of technology that best serve the interests of the people as well as the system are important. Digital technology as a way of training is really benefit. Right?… But it all has to be linked up. (ID28, civil servant/government official)

**3.3 (b) Using digital technologies and data systems – EHR and digital health**	I think digital health will play an important role in facilitating universal health coverage. But you know, digital health can’t be a substitute for the provision of services. So you first have to have services available and focus on those and once that happens, in terms of ensuring the quality of services, it’s also making them transparent in terms of ensuring interoperability in terms of ensuring that there is a longitudinal record available, so that both patients and providers have full information about their health records and so that they will [not] repeat unnecessary diagnostics. (ID15, civil servant/government official)

**3.3 (c) Using digital technologies and data systems – limitations**	I think digital is very good, and frontline workers now they must be trained in its use. They must be given equipment that works well. First, make sure there’s internet connectivity… So to me all the citizen-facing digital things will come slowly to India’s poorer states. So that’s why I’m saying urban areas is where we can do a lot of things […]. It is a very different equation than in a rural areas… So there is disparity and the digital disparity in terms of where you live, if you live in a remote area. (ID28, civil servant/government official)

**3.4 (a) Improving regulation and decentralization**	So what happens is actually a vicious circle, that if you don’t have the capacity, you can’t utilize it. And you don’t apply this capacity. Also, there is an issue of lack of flexibility, because if you are giving funds to states which are divided into say 900 subjects and you don’t have the flexibility of shifting money from one subject to another subject. So that of course is a recipe for non-utilization because by definition, in some of the surveys, you will not be able to use money. Therefore, giving more flexible, strengthening capacity, strengthening the capacity to absorb resources, together with some accountability and monitoring mechanism. (ID15, civil servant/government official)

**3.4 (b) Improving regulation and decentralization – role of central leadership**	The center [needs] to take…on…the role of ensuring that there are regulatory structures again…they will be you know regulatory standards and so on which are centralized mechanisms which are there, and yet [they] allow the flexibility on state levels, depending on the level of development that’s happened in the state itself… But they could lay out some basic principles and mechanisms that must be instituted. It [has] shown enough capacity to take strong-arm action when it wants to, certainly therefore [this is] an area where it should show that. (ID34, civil society organization)


#### 3.1 Strategies for health financing

Most strategies for reforming health financing can be grouped into resource allocation, pooling, and purchasing. More budgetary allocation to health through GHE was perceived as critical to reducing OOPE at the point of care. Making comparisons with other countries that have made significant progress on UHC, policy actors highlighted the need to increase GHE from the historical average of 1.2% to between 2.5 and 5% of GDP. While increasing GHE was near-universally acknowledged, some respondents emphasized the priority to focus on the efficient use of existing resources, especially in the public sector. A few respondents cited examples of Indian states where per capita GHE was estimated to be sufficient for UHC but were still underperforming on health system outcomes. Notwithstanding inefficiencies in resource use, some respondents underscored that inefficiency must not impede additional resource allocation (see Table 3, quote 3.1 (a)).

Almost all policy actors across our four categories favored prepayment and risk pooling to eliminate, or at least reduce, OOPE at the point of care and CHE. Respondents highlighted the need for larger, integrated risk pools that are more likely to be equitable, efficient, and financially viable in the long run. Some respondents mentioned that while OOPE might be inevitable and not necessarily harmful, prepayment and risk pooling would help address uncertainties and CHE (see Table 3, quote 3.1 (b)). Integration of risk pools to address fragmentation was mentioned as a necessary condition to achieve UHC; although none of our respondents offered clear strategies to achieve such integration.

Policy actors recommended mixed financing mechanisms and a combination of sources for resource mobilization, including direct budget allocations and all three major types of insurance mechanisms – tax-financed government health insurance schemes (GHIS), employment-based social health insurance (SHI), and commercial health insurance (CHI). Respondents recommended the need to extend insurance coverage both in terms of population and benefits. Diverse strategies for these included expanding existing GHIS programs with contributory premiums for middle-income populations, introducing SHI for formal sector employees, and designing more inclusive commercial CHI plans. However, some policy actors raised the challenges of implementing these strategies given the limited fiscal space in India to increase coverage and the large informal economy in India that makes it harder to collect premiums or have SHI programs. Some innovative solutions suggested to address these were to link premium collections from the informal sector through utility payments like electricity or mobile phone bills (see Table 3, quote 3.1 (c)). The role of CHI was a point of contention among respondents – very few respondents offered CHI as a potential health financing strategy to achieve UHC, while others raised concerns about adverse selection and cream-skimming associated with voluntary and commercial insurance.

One of the most common recommendations for improving FRP was expanding the benefits package of national insurance to include outpatient services. Policy actors cited the high proportion of OOPE incurred on outpatient care, especially medicines, and diagnostics, and suggested that these health services be covered by insurance. The need to cover a wider range of health conditions, considering the rising burdens of chronic diseases and mental health needs, was also highlighted (see Table 3, quote 3.1 (d)). One of the key themes that emerged under benefits design was the need to target “essential services.” Considering the fiscal constraints, respondents acknowledged that covering all health services for all people is not feasible in the near future. In order to ensure allocative efficiency of scarce government resources, respondents recommended creating a well-defined essential services package, covering aspects of preventive, primary, and hospital care, that is available to the entire population (see Table 3, quote 3.1 (e)).

Within financing reforms, changing how services are purchased or PPMs are structured emerged as a key set of strategies to address poor quality, inefficiencies, and fragmentation. Several policy actors mentioned the need to adopt strategic purchasing – moving away from restrictive and passive methods of line-item budgets and automatic salary payments to more “active” PPMs like outcome-based payments. Two of the most commonly recommended PPMs were capitated payments for primary care facilities based on population enrolment and global budgets for hospitals (see Table 3, quote 3.1 (f))

#### 3.2 Strategies for the organization of healthcare delivery

For the organization of healthcare delivery, the most common theme was the need to consider both the public and private sectors in designing reforms. Our data showed near-universal acknowledgment that India must leverage the large and heterogeneous private sector that caters to the majority of patient encounters. While most respondents mentioned regulation as the strategy to manage the private sector, some listed other strategies like price-setting and PPMs for GHIS-empaneled providers, division of services, and encouraging competition between public and private sector providers (see Table 3, quote 3.2 (a)).

The next most commonly recurring recommendation was about HRH. Policy actors commonly cited the need to move away from a pure headcount and ratio-based approach for planning HRH to a more nuanced and strategic approach to role definitions, efficient HRH allocations, and most importantly, improving the quality of HRH. Related to this, respondents suggested the need to move away from the historical physician-centric model in India to task shifting, where non-physicians could be better suited to expand primary care and UHC. Some respondents mentioned the need to introduce a new cadre of personnel who could provide basic primary care services, while others recommended training and leveraging existing personnel like practitioners of Indian systems of medicine or Ayush practitioners, private pharmacists, solo practitioners with clinical qualifications, and even informal providers (see Table 3, quote 3.2 (b)).

Policy actors recommended reorganizing the health delivery system to introduce gatekeeping to address bypassing of primary care facilities and improving referral linkages between levels of care and even between some private sector providers (like pharmacists and solo practitioners) to secondary and tertiary level facilities. These recommendations were closely linked to using strategic purchasing as a policy lever and implementing digital technologies to connect providers and patients (see Table 3, quote 3.2 (c)).

#### 3.3 Using digital technologies and data systems

The use of digital health technologies was one of the most recurring reform ideas that emerged. Policy actors suggested digital technologies to deliver better quality care and address gaps in access. Digital technologies were also recommended to integrate different levels of care, different types of providers and improve referral linkages, all aimed at reducing fragmentation in India’s health delivery system. Digital systems were also suggested to address inefficiencies in health financing and resource allocation and to monitor quality for outcome-based payments. Most participants spoke about the need for a nationally linked health record system across states, between public and private sectors, and throughout different levels of care down to the community (see Table 3, quote 3.3 (a)). However, some respondents cautioned against considering digital technologies as the “magic bullet” that will fix a broken health system and raised concerns about the inequitable access to technologies in many parts of the country and by several disadvantaged communities (see Table 3, quote 3.3 (b)).

#### 3.4 Improving regulation and decentralization

Several policy actors emphasized the need for decentralized decision-making, program design, and implementation of health policies. To address the diversity of state-specific needs in India, respondents recommended that state governments lead the design of health reforms and that decision-making be decentralized to districts, blocks, and villages. Higher financial autonomy for decentralized governments through increased state-GHE and structuring more flexible federal government budgetary allocations were key strategies (see Table 3, quote 3.4 (a)). In contrast, some respondents raised concerns about the ability of decentralized levels to design and lead reforms, given the variable state capacity in the country. They perceived that decentralization might lead to more inter-state inequities as higher-resourced states would have better systems to achieve UHC. Some of the policy actors highlighted the critical role of the national government and underscored that stewardship for overall health system design, and that the national government must lead regulations that are core to advancing UHC (see Table 3, quote 3.4 (b)).

## Discussion and Conclusion

Using the CKF and inductive analysis, this study presents the conceptualization of UHC, the barriers they perceive as impeding India’s progress toward UHC, and the policy reforms they suggest to address these barriers, as voiced by 38 leading policy actors in India. Our data show broad consensus among policy actors on the meaning of UHC. With the exception of some specific aspects of higher emphasis, we did not find contrasting views on UHC and health system challenges among the different groups of policy actors from across research and academic institutions, civil society, private sector, and government. This finding is congruent with other research from India [14], but contrasts with global evidence showing marked differences in conceptualizations among stakeholders [15,16,17].

Most of the policy actors’ perceptions about UHC and the conceptualizations of the challenges were congruent with the widely accepted definitions of UHC and the extant literature on problem analysis of UHC goals in India. For example, low GHE and high OOPE have been widely accepted as longstanding problems with India’s health system [18,19]. Although recent data shows a decline, these are still far below peer averages [6]. Similarly, although under-researched, poor quality of care is recognized as a pervasive problem in the country [5,20,21]. Other areas of consensus were the lack of a comprehensive systems approach to health reforms over the last several decades and a fragmentation of the system, especially between public and private sectors, which were unanimously identified as barriers to UHC.

Drawing from the CKF, the levers of health financing, organization of healthcare delivery, provider payment, and regulation were near-universally listed as strategies to achieve UHC. Our inductive analysis reveals the use of digital technologies and decentralization as two other strategies outside the CKF. Similar priority areas for reforms were reported from other countries, including the importance of addressing fragmentation through coordinated policies for the public and private sectors, addressing financial risk protection through expanding insurance, and bringing about strategic purchasing and provider payment reforms [22,23,24,25]. Despite significant consensus on the barriers and reform areas, our data revealed important areas of contention regarding the policy changes. In particular, our respondents differed on the role of commercial insurance in UHC, using digital technologies in service delivery, and the emphasis on the efficient use of existing resources versus additional investments for health. The series of stakeholder workshops reported similar contentions on the theory of change for UHC in India [14], as well as by studies from other low and middle-income countries with mixed health systems that are transitioning toward health system reforms for UHC [16,22,26,27]. These differences in points of view set the stage for future research.

Further, our findings show that while most policy actors agreed on the nature of the barriers to UHC, they did not suggest complementary strategies to address these barriers. Thus, while three areas – poor quality of care, engagement with the private sector for UHC, and the lack of a comprehensive systemic approach to health policies – were the most commonly cited UHC goals, very few explicit strategies were offered to achieve them. Instead, most respondents broadly mentioned the use of regulations to improve quality and engage the private sector. However, evidence from other mixed health systems shows that regulation is only one option out of many others – such as prohibition, encouragement, and purchasing – to effectively engage the private sector [28]. In fact, research, including from India, shows that regulation has been largely ineffective as a strategy owing to weak state capacity for enforcement, poor governance structures, and corruption and regulatory capture [5,29,30,31,32]. Policy actors’ vision for the respective roles of the public and private sectors in India’s health system was also a major area of contention in our findings. These may indicate why the poor quality of care and a lack of meaningful private sector engagement have persisted despite being recognized as critical issues.

The contrasting points of view among the policy actors reflect the debates in India over the last several decades. Many challenges and policy solutions mentioned by the interviewees are congruent with the key policy documents like the High-Level Expert Group (HLEG), the Lancet Series on UHC in India, and the National Health Policy 2017 [33,34,35]. However, we also see a significant diversity in opinions and new emerging themes. Notably, digital technologies, health system reforms, and health financing are far more common themes in our data, as is the acknowledgment of the need to involve the private sector in UHC reforms. Our study sample included a broader range of stakeholders from various backgrounds compared to the committees that have drafted key policy documents in India in the past. This might explain the diversity of opinions, especially about policy solutions to achieve UHC. This diversity of viewpoints aligns with the Lancet Commission’s objective of convening and representing a wide range of stakeholders.

Our data indicated some path dependency in the strategy recommendations. There were very few strategies for transformative and comprehensive systemic reforms. Most suggestions were limited to one or two policy levers without considering the inevitable consequences for other levers in the health system – almost tending towards the prevailing vertical approaches that respondents have themselves critiqued. These findings underscore the need to recognize the complex and interlinked nature of health system reforms and initiate a path-departure to bring about transformation – the core goal of the Commission.

A conspicuously infrequent theme in our data was the politics of healthcare and health reforms. Although there is recognition that health reforms are inherently political processes, there has been minimal research on these topics [36,37,38,39,40,41]. To course-correct and increase the chances of success, analyses of agenda-setting in health and examining why specific issues like quality or certain reforms like regulations have consistently underperformed could lend valuable insights.

Our study has several limitations. First, although our sample had a diverse representation of policy actors, they were selected through convenience and snowball methods and might have been affected by selection biases. However, we could not identify any more appropriate sampling procedure for this specific population. While we continued sampling until thematic saturation was reached, our findings might not necessarily reflect the views of all policy actors in India, especially politicians. Like most research with policy elites, our findings only represent the perspective of a very small, privileged, and influential group of stakeholders in the UHC landscape. There are several other stakeholders, like members of the public or healthcare providers, whose views on UHC might be materially different from those of our respondents. We are addressing this by undertaking two other large-scale surveys of households’ and physicians’ perceptions of UHC as a part of the Lancet Commission. Finally, we reflect on our positionality in interpreting the data. Four of the authors have lived and worked in India; the first and senior authors of this study have been closely engaged with several UHC efforts, health reforms, and expert groups in India. As such, many of the health system gaps and barriers are lived experiences for them. One of the authors has primarily worked in high-income country settings, and their professional and lived experiences might have added an “outsider’s” lens to our analysis and interpretations.

Notwithstanding these limitations, our study is the first systematic examination of a diverse set of policy actors’ problem analyses and suggestions for health system reforms to advance UHC goals in India. The study offers insights into why certain acknowledged problems in India’s health system have not seen any transformative change or have faced path dependencies. Future areas of research could be to examine the reasons behind low prioritization and identify strategies for reforms in certain areas, such as quality of care, private sector engagement, and the political analysis of health reforms. This qualitative study has been used for hypothesis formation; our findings have informed a large national physicians’ survey in India to elicit their views about UHC. The findings from this study will be triangulated with other primary research undertaken by the Commission, including the physicians’ survey, a nationally representative population survey to elicit citizen preferences and experiences of the health system, and case studies of districts purposively selected to reflect a range of performance indicators on a newly developed UHC index; these will also be interpreted in light of the assumptions laid out by the Commission’s theory of change workshops [14]. Together, this body of work will inform the Commission’s problem analyses and recommendations for health system reforms needed to realize UHC over the next ten years.
